# *Trans*-Anethole Alleviates Trimethyltin Chloride-Induced Impairments in Long-Term Potentiation

**DOI:** 10.3390/pharmaceutics14071422

**Published:** 2022-07-06

**Authors:** Wonseok Chang, Jihua An, Geun Hee Seol, Seung Ho Han, Jaeyong Yee, Sun Seek Min

**Affiliations:** 1 Department of Physiology and Biophysics, School of Medicine, Eulji University, Daejeon 34824, Korea; cws2017@eulji.ac.kr (W.C.); jinhua221@hanmail.net (J.A.); hans0424@eulji.ac.kr (S.H.H.); 2 Department of Basic Nursing Science, School of Nursing, Korea University, Seoul 02841, Korea; ghseol@korea.ac.kr

**Keywords:** *trans*-anethole, trimethyltin chloride, long-term potentiation (LTP), synaptic plasticity, hippocampus

## Abstract

*Trans*-anethole is an aromatic compound that has been studied for its anti-inflammation, anticonvulsant, antinociceptive, and anticancer effects. A recent report found that *trans*-anethole exerted neuroprotective effects on the brain via multiple pathways. Since noxious stimuli may both induce neuronal cell injury and affect synaptic functions (e.g., synaptic transmission or plasticity), it is important to understand whether the neuroprotective effect of *trans*-anethole extends to synaptic plasticity. Here, the effects of trimethyltin (TMT), which is a neurotoxic organotin compound, was investigated using the field recording method on hippocampal slice of mice. The influence of *trans*-anethole on long-term potentiation (LTP) was also studied for both NMDA receptor-dependent and NMDA receptor–independent cases. The action of *trans*-anethole on TMT-induced LTP impairment was examined, too. These results revealed that *trans*-anethole enhances NMDA receptor-dependent and -independent LTP and alleviates TMT-induced LTP impairment. These results suggest that *trans*-anethole modulates hippocampal LTP induction, prompting us to speculate that it may be helpful for improving cognitive impairment arising from neurodegenerative diseases, including Alzheimer’s disease.

## 1. Introduction

**Figure 1:**
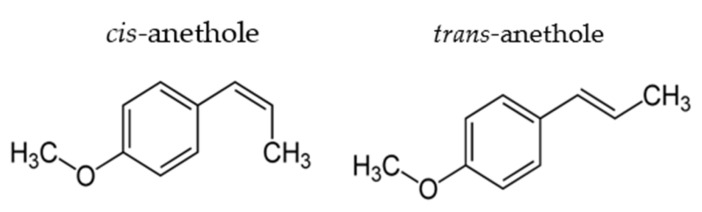
Chemical structural formula of *cis*- and *trans*-anethole.

Anetholes, which exists in the *cis* and *trans* forms, are widely used as a flavor substance (Figure 1)*. Trans*-anetholes are more abundant in essential oil (trans/cis ration of about 9 to 1) [1] and preferred for use. *Cis*-anetholes are considered potentially toxic for humans [2]. *Trans*-anethole, which is the main component of fennel oil [3], is an aromatic compound that is also widely used in commercial medicines. Previous work showed that *trans*-anethole has anti-inflammation, anticonvulsant, antinociceptive, and anticancer capacities [4,5,6,7]. *Trans*-anethole was also reported to have an anti-amnesic effect in behavioral tasks [8] and to exert a neuroprotective effect in cortical neuronal cells [9]. However, the potential effects of *trans*-anethole are not fully understood and its action mechanism(s) in the nervous system have not been elucidated.

One of the potential effects of *trans*-anethole that warrants further study is its potential protective effect against the action of organometallic compounds. Such actions have gained wide interest recently given findings on environmental pollution with organometallic compounds and their association with health impairments, such as Alzheimer’s disease (AD). The organometallic compound trimethyltin chloride (TMT) is known to exert neurotoxicity in the cerebral cortex and hippocampus [10,11,12] and induce learning and memory impairments similar to those of AD in animal models [13,14,15]. Mechanistically, TMT alters the expression of the amyloid precursor protein, presenilin 1, and other factors that play central roles in the pathophysiology of AD [14,15]. Given these findings, TMT-induced dementia is considered to be an experimental model of AD [16,17].

Long-term potentiation (LTP), which is one of several phenomena underlying synaptic plasticity, is widely considered to be a major cellular mechanism of learning and memory in neuroscience [16]. Mechanistically, there are two reported types of LTP. Referred to as NMDA receptor-dependent and -independent LTP, the two forms differ in how they are triggered in an experimental setting. NMDA receptor-dependent LTP is normally achieved by applying lower-frequency tetanic stimulation and can be almost fully blocked by NMDA receptor antagonists [17,18,19]. NMDA receptor-independent LTP, which can still be observed in the presence of these agents [20,21], is experimentally induced by the application of a chemical, such as tetraethylammonium chloride (TEA) [22,23,24,25].

The aim of this study was to investigate the enhancement and/or protective effects of *trans*-anethole on LTP induction in mouse hippocampus. The present study tested the effects of *trans*-anethole on the basal inductions of NMDA-dependent and -independent LTPs, confirmed that TMT impairs the induction of LTPs, and investigated the effects of *trans*-anethole on the TMT-induced impairment of LTP induction.

## 2. Materials and Methods

### 2.1. Experimental Animals

Male and female C57BL/6 mice (three to six weeks of age when experiments commenced) were used. All animals were housed in a temperature-controlled room (22–25 °C) under a 12-h light/dark cycle in which the lights-on period began on at 07:00. Food and water were available ad libitum. All experiments were approved by the Institutional Animal Care and Use Committee of Eulji University (EUIACUC19-27).

### 2.2. Preparation of Hippocampal Slices

The procedures used for the electrophysiological experiments were as described previously [26,27]. Briefly, mice were decapitated under deep isoflurane anesthesia and brains were quickly removed in ice-cold dissection buffer containing sucrose (212.7 mM), KCl (2.6 mM), NaH_2_PO_4_ (1.23 mM), NaHCO_3_ (26 mM), dextrose (10 mM), MgCl_2_ (10 mM), and CaCl_2_ (0.5 mM). Horizontal brain sections (400 μm thick) were prepared using a vibratome (Campden Instruments; Loughborough, UK) and placed in dissection buffer that was continuously bubbled with 95% O_2_/5% CO_2_ (*v*/*v*). The slices were held at 35 °C for 1 h in a chamber filled with continuously oxygenated artificial cerebrospinal fluid (ACSF) of the following composition: NaCl (124 mM), KCl (5 mM), NaH_2_PO_4_ (1.25 mM), NaHCO_3_ (26 mM), dextrose (10 mM), MgCl_2_ (1.5 mM), and CaCl_2_ (2.5 mM). The slices were then transferred to a submersion recording chamber that was maintained at 30 °C, and perfused with oxygenated ACSF at a flow rate of 2 mL/min.

### 2.3. Electrophysiological Recordings

A bipolar stimulating electrode was inserted into the stratum radiatum to activate the Schaffer collaterals of CA1 pyramidal cells. A glass micropipette filled with ACSF was inserted into the CA1 pyramidal layer to record field potentials (FPs). CA1 FPs were evoked by stimulating the Schaffer collaterals with electrical pulses of 2 ms in duration, delivered with the aid of concentric bipolar stimulating electrodes (FHC; Bowdoinham, ME, USA). The initial slopes of extracellular FPs were recorded in the CA1 stratum radiatum. Baseline responses were obtained upon application of 50% of the maximal stimulation, at 0.033 Hz. LTP was induced using electrical stimulation or chemical application paradigms. For the NMDA receptor-dependent protocol, electrical stimulation was supplied by one episode of theta-burst stimulation (1 TBS; protocol consisted of eight bursts of four 100-Hz pulses administered at 200-ms intervals). The stimulus intensity during TBS was identical to that of the test pulse. For NMDA receptor-independent LTP, the K^+^ channel blocker, tetraethylammonium (TEA) was applied. All measurements are expressed as percentages of the average values calculated 20 min prior to LTP induction. Significant differences between groups were assessed by evaluating the average LTP values for 58–60 min after LTP induction. To measure paired-pulse facilitation (PPF), inter-stimulus intervals (ISIs) of 25 ms, 50 ms, 100 ms, 200 ms, 400 ms, 1000 ms, and 2000 ms were used.

### 2.4. Drugs

*Trans*-anethole, trimethyltin chloride (TMT), tetraethylammonium chloride (TEA), and *DL*-APV were purchased from Sigma (St. Louis, MO, USA). *Trans*-anethole was dissolved in alcohol and the other drugs were dissolved in distilled water.

### 2.5. Data Analysis

Data analysis was performed using IBM SPSS Statistics 21 (SPSS Inc.; Chicago, IL, USA). All values are given as means ± SEMs; the error bars in the figures also represent SEMs. Statistical significance was assessed using Student’s *t*-test or one-way ANOVA, followed by Tukey HSD testing. Probability values *p* < 0.05 were considered statistically significant.

## 3. Results

### 3.1. Trans-Anethole Enhances NMDA Receptor-Dependent LTP

**Figure 2:**
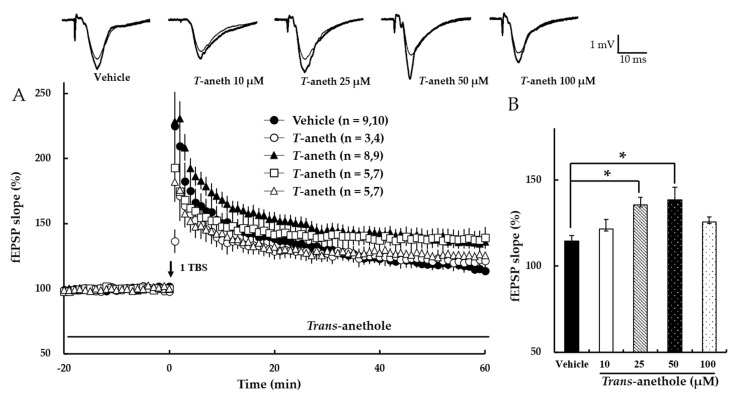
*Trans*-anethole enhances NMDA receptor-dependent LTP. (**A**) NMDA receptor-dependent LTP was induced by 1 TBS. *Trans*-anethole was applied during the time period indicated by the bar. The numbers of studied mice and slices, respectively, are presented in parenthesis. The filled circles, open circles, filled triangles, open squares, and open triangles indicate the Vehicle group, 10 µM *Trans*-anethole group, 25 µM *Trans*-anethole group, 50 µM *Trans*-anethole group, and 100 µM *Trans*-anethole group, respectively. (**B**) Values represent the percent change observed in the fEPSP slope at 58–60 min after 1 TBS. Statistical significance (* *p* < 0.05 vs. Vehicle) was calculated by one-way ANOVA followed by Tukey HSD test. Examples of responses recorded 1 min before (thin traces) or 60 min after (thick traces) TBS are shown above. * *p* < 0.05 vs. the corresponding Vehicle group. *T*-aneth, *Trans*-anethole; fEPSP, field excitatory synaptic potential.

We first investigated the effects of *trans*-anethole on NMDA receptor-dependent LTP induced by one episode of TBS (1 TBS). *Trans*-anethole did not affect the baseline field excitatory postsynaptic potential (fEPSP) value at any tested dose (data not shown). In contrast, *trans*-anethole dose-dependently enhanced NMDA receptor-dependent LTP at up to 50 µM (Figure 2). Figure 2B presents a bar diagram showing the average LTP values obtained at 58–60 min after 1 TBS (Vehicle: 115 ± 3%, 10 µM *Trans*-anethole: 121 ± 6%, 25 µM *Trans*-anethole: 135 ± 5%, 50 µM *Trans*-anethole: 138 ± 9%, 100 µM *Trans*-anethole: 126 ± 3%; * *p* < 0.05). These results suggest that *trans*-anethole enhances NMDA receptor-dependent LTP.

### 3.2. Trans-Anethole Enhances NMDA Receptor-Independent LTP

**Figure 3:**
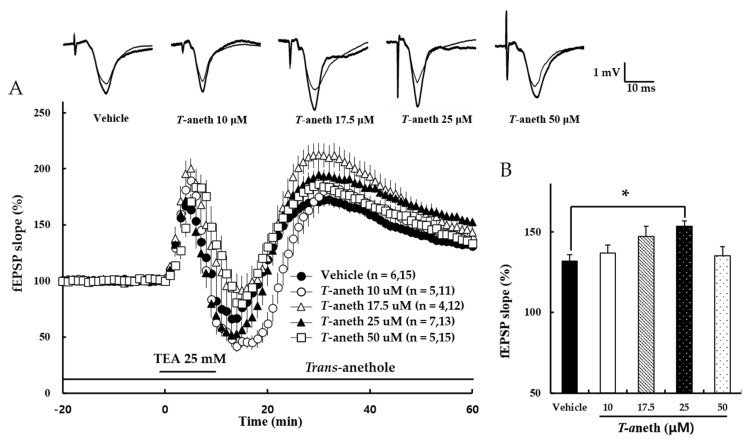
*Trans*-anethole (25 µM) enhances NMDA receptor-independent LTP. (**A**) NMDA receptor-independent LTP was induced by TEA (25 mM), which was applied during the time period indicated by the bar and then washed out. The numbers of mice and slices, respectively, are shown in parentheses. Filled circles, open circles, open triangles, filled triangles, and open squares indicate the Vehicle group, 10 µM *Trans*-anethole group, 17.5 µM *Trans*-anethole group, 25 µM *Trans*-anethole group, and 50 µM *Trans*-anethole group, respectively. (**B**) Values represent percent changes in the fEPSP slope observed at 58–60 min after TEA exposure. Statistical significance (* *p* < 0.05 vs. Vehicle) was calculated by one-way ANOVA followed by Tukey HSD test. Typical traces of fEPSPs recorded 1 min before (thin traces) or 60 min after (thick traces) TBS are shown above. *T*-aneth, *Trans*-anethole; fEPSP, field excitatory synaptic potential.

Next, the effect of *trans*-anethole on NMDA receptor-independent LTP was tested. Here, LTP was induced by tetraethylammonium (TEA), one of the K^+^ channel blocker. Under a stable basic transmission, TEA (25 mM) was applied for 10 min and then washed out (Figure 3A). Our results revealed that *trans*-anethole dose-dependently enhanced NMDA receptor-independent LTP. Figure 3B presents a bar diagram showing the average LTP values obtained at 58–60 min after TBS (Vehicle: 132 ± 4%, 10 µM *Trans*-anethole: 137 ± 3%, 17.5 µM *Trans*-anethole: 147 ± 6%, 25 µM *Trans*-anethole: 154 ± 4%, 50 µM *Trans*-anethole: 135 ± 6%; F_(4,62)_ = 3.21; * *p* < 0.05). These results suggest that *trans*-anethole enhances NMDA receptor-independent LTP.

### 3.3. NMDA Receptor Antagonist Treatment Does Not Alter TEA-Induced LTP Induction

**Figure 4:**
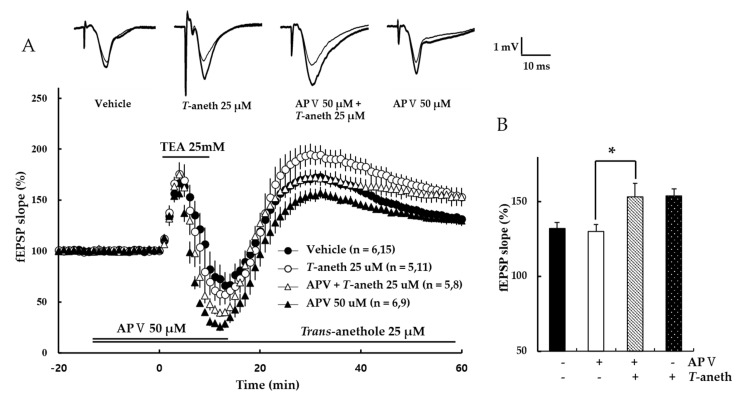
An NMDA receptor antagonist does not affect TEA-induced LTP induction. (**A**) NMDA receptor-independent LTP was induced by TEA. APV (50 µM) and TEA (25 mM) were applied during the time periods indicated by the bars, and then washed out. (**B**) Values represent percent changes observed in the fEPSP slope at 58–60 min after TEA exposure. The numbers of mice and slices, respectively, are shown in parentheses. Filled circles, open circles, open triangles, and filled triangles indicate the Vehicle group, 25 µM *Trans*-anethole group, 50 µM APV + 25 µM *Trans*-anethole group, and 50 µM APV group, respectively. Statistical significance (* *p* < 0.05 vs. Vehicle) was calculated by one-way ANOVA followed by Tukey HSD test. *T*-aneth, *Trans*-anethole; APV, *DL*-APV; fEPSP, field excitatory synaptic potential.

To confirm that TEA-induced LTP is NMDA receptor-independent, the NMDA receptor antagonist, APV, was applied for 30 min and then used TEA for a wash out. Our results revealed that TEA induced LTP in the presence of APV (Vehicle: 132 ± 4%, 50 µM APV: 130 ± 5%, 50 µM APV + 25 µM *trans*-anethole: 153 ± 9%, 25 µM *trans*-anethole: 154 ± 4%, *p* < 0.05; Figure 4). This suggests that TEA induces NMDA receptor-independent LTP.

### 3.4. Trans-Anethole Does Not Affect the Paired-Pulse Facilitation Ratio

**Figure 5:**
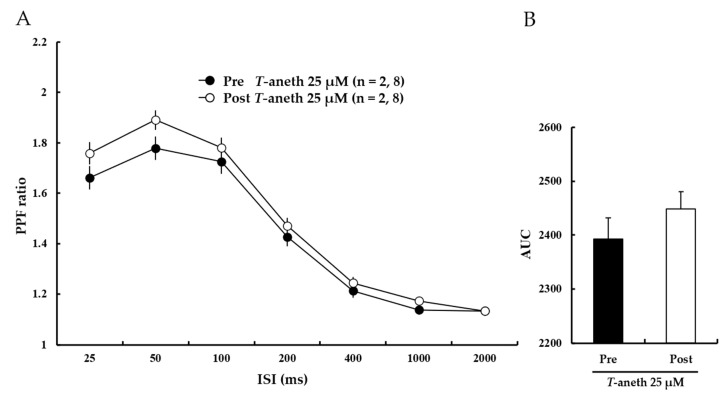
Anethole (25 µM) treatment does not affect the PPF ratio. (**A**) Paired-pulse facilitation (PPF) ratios were similar before and after perfusion of anethole (25 µM). Filled circles and open circles indicate the Pre-25 µM *Trans*-anethole group and Post-25 µM *Trans*-anethole group, respectively. (**B**) The areas under the curve (AUCs) for the PPF ratio did not significantly differ. Statistical significance (Post-*Trans*-anethole group vs. Pre-*Trans*-anethole group) was calculated by Student’s *t*-test. ISI: interstimulus interval, AUC: area under the curve. *T*-aneth: *Trans*-anethole.

The paired-pulse facilitation (PPF) ratio was further investigated to identify if the enhancing effect of *trans*-anethole on LTP induction was attributable to changes in presynaptic transmission. The results showed that treatment with *trans*-anethole (25 μM) did not significantly alter the PPF ratio (Figure 5A). The bar graph presented in Figure 5B shows the areas under the curve (AUCs) for the PPF ratios (Pre-25 μM *trans*-anethole: 2393 ± 39%, Post-25 μM *trans*-anethole: 2448 ± 32%). These results suggest that *trans*-anethole enhances LTP induction through changes in post-synaptic transmission.

### 3.5. Trimethyltin Chloride (TMT) Dose-Dependently Impairs NMDA Receptor-Dependent LTP

**Figure 6:**
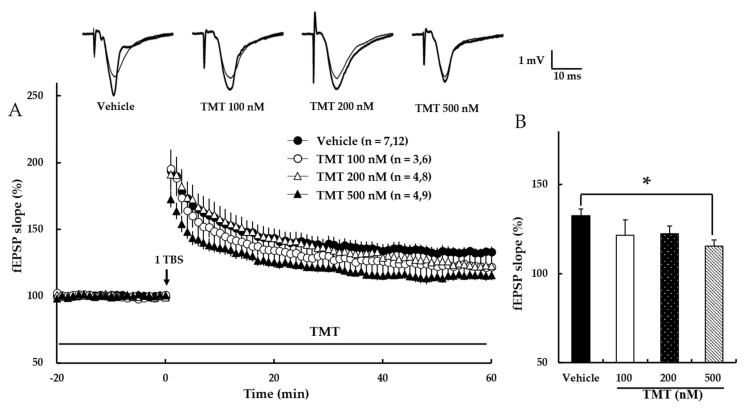
TMT reduces NMDA receptor-dependent LTP in a dose-dependent manner. (**A**) The responses represent LTP induction over 1 h after NMDA receptor-dependent LTP was induced by 1 TBS. TMT in aCSF solution was applied at the beginning of the recording and perfused throughout the time period indicated by the bar. The numbers of mice and slices, respectively, are expressed in parentheses. Filled circles, open circles, open triangles, and filled triangles indicate the Vehicle group, 100 nM TMT group, 200 nM TMT group, and 500 nM TMT group, respectively. (**B**) Values represent percent changes in the fEPSP slope observed at 58–60 min. Statistical significance (* *p* < 0.05 vs. Vehicle) was calculated by one-way ANOVA followed by Tukey HSD test.; fEPSP: field excitatory synaptic potential.

TMT reportedly exhibits neurotoxicity, especially in the hippocampus. To evaluate the effect of TMT on hippocampal synaptic plasticity, NMDA receptor-dependent LTP was investigated. Our results revealed that TMT dose-dependently reduced NMDA receptor-dependent LTP (Vehicle: 133 ± 4%, 100 nM TMT: 122 ± 8%, 200 nM TMT: 122 ± 4%, 500 nM TMT: 116 ± 4%; *p* < 0.05; Figure 6), suggesting that TMT impairs NMDA receptor-dependent LTP.

### 3.6. TMT Does Not Affect the PPF Ratio

**Figure 7:**
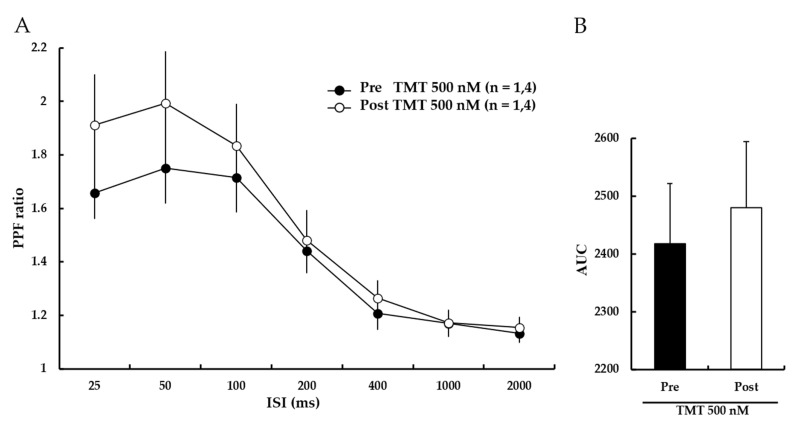
TMT (500 nM) does not affect the PPF ratio. (**A**) PPF ratios were similar before and after TMT (500 nM) perfusion. Filled circles and open circles indicate the Pre-500 nM TMT group and Post-500 nM TMT group, respectively. (**B**) The AUCs for the PPF ratio were not significantly different. Statistical significance (Post-TMT group vs. Pre-TMT group) was calculated by Student’s *t*-test. ISI: interstimulus interval, AUC: area under the curve.

Next, PPF ratios were measured to investigate whether the ability of TMT to reduce LTP was attributable to changes in presynaptic transmission. The results showed that the PPF ratio was not significantly altered by TMT (500 nM) treatment (Figure 7A). The bar graph presented in Figure 6B shows the AUCs for the PPF ratios (Pre-500 nM TMT: 2418 ± 104%, Post-500 nM TMT: 2480 ± 115%). These results suggest that TMT reduces LTP induction through changes in post-synaptic transmission.

### 3.7. Trans-Anethole Blocks the TMT-Induced Impairment in NMDA Receptor-Dependent LTP

**Figure 8:**
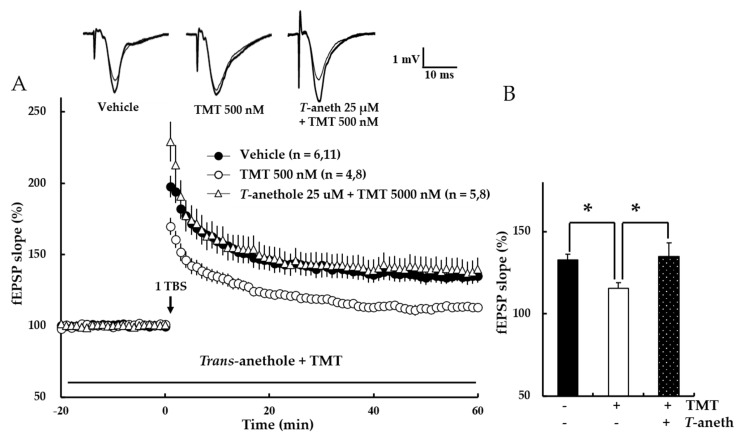
*Trans*-anethole (25 µM) attenuates TMT-induced impairment in NMDA receptor-dependent LTP. (**A**) The responses represent LTP induction over 1 h after NMDA receptor-dependent LTP was induced by 1 TBS. *Trans*-anethole (25 µM) was applied 10 min before the addition of TMT (500 nM), which was perfused in aCSF during the time period indicated by the bar. The numbers of mice and slices, respectively, are shown in parentheses. Filled circles, open circles, and open triangles indicate the Vehicle group, 500 nM TMT group, and 25 µM *Trans*-anethole + 500 nM TMT group, respectively. (**B**) Values represent percent changes in the EPSP slope observed at 58–60 min. Statistical significance (* *p* < 0.05 vs. 500 nM TMT) was calculated by one-way ANOVA followed by Tukey HSD test. *T*-aneth: *Trans*-anethole; fEPSP: field excitatory synaptic potential.

To measure the effect of *trans*-anethole on TMT-induced LTP impairment, NMDA receptor-dependent LTP was first investigated. In this experiment, *trans*-anethole (25 µM) was applied 10 min before the application of TMT (500 nM). The results revealed that *trans*-anethole blocked the ability of TMT to impair 1 TBS-induced LTP (Vehicle: 134 ± 3%, 500 nM TMT: 113 ± 3%, 25 µM *Trans*-anethole + 500 nM TMT: 138 ± 8%; * *p* < 0.05; Figure 8). These results suggest that *trans*-anethole blocks the TMT-induced impairment of NMDA receptor-dependent LTP.

### 3.8. Trans-Anethole Blocks TMT-Induced NMDA Receptor-Independent LTP Impairment

**Figure 9:**
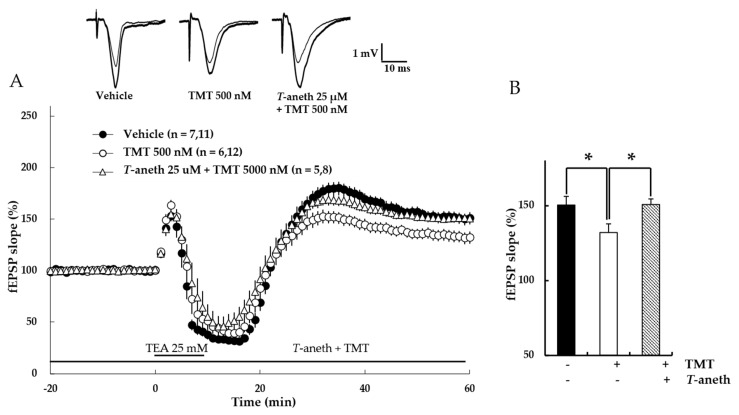
*Trans*-anethole (25 µM) modulates the TMT-induced impairment of NMDA receptor-independent LTP. The responses represent LTP induction at 1 h after 1 TBS (**A**). NMDA receptor-independent LTP was induced by TEA. *Trans*-anethole (25 µM) was applied 10 min before TMT (500 nM). Each drug was exposed during the time period indicated by the corresponding bar (**B**). Values represent percent changes in the fEPSP slope observed at 58–60 min (**B**). The numbers of mice and slices, respectively, are expressed in parentheses. Filled circles, open circles, and open triangles indicate the Vehicle group, 500 nM TMT group, and 25 µM anethole + 500 nM TMT group, respectively. Statistical significance (* *p* < 0.05 vs. 500 nM TMT) was calculated by one-way ANOVA followed by Tukey HSD test. *T*-aneth: *Trans*-anethole; fEPSP: field excitatory synaptic potential.

To measure the effects of *trans*-anethole on TMT-induced LTP impairment, NMDA receptor-independent LTP was investigated. In this experiment, LTP was induced by the K^+^ channel blocker, TEA. Our results revealed that *trans*-anethole alleviated the TMT-induced impairment in TEA-induced LTP (Figure 9A). In the presence of 25 µM *trans*-anethole, there was no TMP-induced LTP impairment (Vehicle: 150 ± 6%, 500 nM TMT: 132 ± 6%, *Trans*-anethole + 500 nM TMT: 151 ± 4%; * *p* < 0.05; Figure 9B). These results suggest that *trans*-anethole blocks the TMT-induced impairment of NMDA receptor-independent LTP.

## 4. Discussion

This study demonstrates that *trans*-anethole enhances NMDA receptor-dependent and -independent LTP induction, TMT impairs LTP induction, and *trans*-anethole modulates TMT-induced LTP impairment. Hippocampal LTP, which is one of several phenomena underlying synaptic plasticity, is widely considered to be a major cellular mechanism of learning and memory in neuroscience [16]. Hippocampal LTP is generally classified as NMDA receptor-dependent or -independent LTP, and these types of LTP were induced in the present study by applying 1 TBS or the K^+^ channel blocker, TEA, respectively. One TBS-induced LTP is widely considered a representative NMDA receptor-dependent LTP, and can be largely blocked by NMDA receptor antagonists [17,18,19]. TEA-induced LTP requires the activation of voltage-dependent Ca^2+^ channels (VDCCs) for Ca^2+^ influx [22]. Both T-type VDCCs and nifedipine-sensitive L-type VDCCs are reportedly involved in TEA-induced LTP at CA1 [28,29,30]. The previous findings are consistent with our results presented in Figure 3, which shows that TEA-induced LTP induction is not blocked by the NMDA receptor antagonist, *DL*-APV.

The present study showed that *trans*-anethole enhanced both NMDA receptor-dependent and NMDA receptor-independent LTP induction (Figure 2 and Figure 3). This indicates that *trans*-anethole has enhancing effects on LTPs induced by activation of both NMDA receptor and VDCCs. However, the ability of *trans*-anethole to increase LTP showed a tendency to decrease at high concentrations (100 μM and 50 μM in NMDA receptor-dependent and -independent LTP, respectively). We speculate that *trans*-anethole enhances LTP by influencing specific molecules in the induction cascades of NMDA receptor-dependent and -independent LTP. However, we do not yet know which molecule(s) are targeted by *trans*-anethole for this action, and whether higher concentrations of *trans*-anethole trigger a more complex effect involving additional molecules.

Measurement of the PPF ratio is a classical protocol for testing pre-synaptic transmission in the hippocampus. We did not observe a significant difference in the AUCs for the observed PPF ratios after *trans*-anethole treatment, although the individual data points were enhanced (Figure 5). This indicates that *trans*-anethole affects LTP induction through post-synaptic transmission

Our results showed that TMT dose-dependently impaired NMDA receptor-dependent LTP induction (Figure 6). The AUC of the PPF ratio was not significantly altered following TMT treatment (Figure 7). These results are consistent with a previous report indicating that TMT blocks glutamatergic receptor channels [31]. Thus, TMT appears to affect synaptic plasticity by altering post-synaptic transmission and exerting memory impairment via the NMDA receptor.

The present study is the first to demonstrate that *trans*-anethole facilitates normal hippocampal NMDA receptor-dependent and -independent LTP induction. *Trans*-anethole was previously reported to enhance memory capacity in behavioral tasks [8]. Consistent with our results presented in Figure 5, previous reports indicated that TMT can induce neurotoxicity, loss of neuronal cells, and impairments of hippocampal learning and memory assessed using different methods [12,13,15]. Since *trans*-anethole and TMT affected LTP induction via NMDA receptor and VDCCs in normal hippocampus, we speculated that *trans*-anethole should play a positive role in TMT-induced LTP impairment. Indeed, we found that *trans*-anethole positively modulated TMT-induced NMDA receptor-dependent and -independent LTP impairment (Figure 8 and Figure 9). Since both *trans*-anethole and TMT exerted the effects on basic transmission through postsynaptic plasticity (Figure 5 and Figure 7), we speculate that *trans*-anethole may modulate TMT-induced LTP impairments via postsynaptic plasticity. The general finding of this study is that *trans*-anethole modulates the TMT-induced inhibition of NMDA receptor and VDCCs in LTP induction via postsynaptic plasticity.

## 5. Conclusions

The present results demonstrate that *trans*-anethole enhances NMDA receptor-dependent and -independent LTP induction. Present study also show that TMT impairs LTP, and this action is blocked by *trans*-anethole. These results suggest that *trans*-anethole may improve learning and memory and could represent a potential therapeutic. Future work is needed to clarify the mechanisms through which *trans*-anethole acts on normal or TMT-impaired hippocampal LTP.

## Data Availability

Not applicable.
